# Knowledge, attitude, practice, and factors associated with prevention practice towards COVID-19 among healthcare providers in Amhara region, northern Ethiopia: A multicenter cross-sectional study

**DOI:** 10.1371/journal.pgph.0000171

**Published:** 2022-04-11

**Authors:** Wassachew Ashebir, Belete Yimer, Atsede Alle, Muluken Teshome, Yohannes Teka, Awraris Wolde

**Affiliations:** 1 Department of Public Health, Debre Markos University, Debre Markos, Ethiopia; 2 Department of Human Nutrition, Debre Markos University, Debre Markos, Ethiopia; Makerere University School of Public Health, UGANDA

## Abstract

Healthcare providers (HCPs) are at an increased risk of getting COVID-19 as a result of their front-line works. Health behaviors of HCPs can influence prevention and control actions implemented in response to the pandemic. Hence, this study aimed to assess the knowledge, attitude, and practice (KAP) and factors associated with prevention practice towards COVID-19 among healthcare providers in Amhara region, northern Ethiopia. A multicenter cross-sectional study was conducted among 422 HCPs in selected public health facilities of Amhara region, between 20^th^ September and 20^th^ October 2020. Data related to HCP’s KAP and socio-demographic characteristics were collected using a pre-tested self-administered questionnaire. Bloom’s cut-off ≥ 80%, ≥90%, and ≥75% was used to determine adequate knowledge, positive attitude, and good prevention practice, respectively. Data were analyzed using SPSSS version 25.0. A multivariable logistic regression analysis was performed to identify factors significantly associated with COVID-19 prevention practice. Statistical significance was determined at a p-value of < 0.05 and the presence of association was described using odds ratio (OR) with their 95% confidence interval (CI). Overall, 368 (89.8%), 387 (94.4%), and 326 (79.5%) HCPs had adequate knowledge, positive attitude, and good prevention practice towards COVID-19, respectively. Factors significantly associated with good COVID-19 prevention practice were being a Nurse in profession (AOR = 2.13, 95% CI = 1.13–3.99), having < 5 years of working experience (AOR = 0.46, 95% CI = 0.24–0.86), using social media (AOR = 6.20, 95% CI = 2.33–16.51) and television and or radio (AOR = 4.03, 95% CI = 1.56–10.38) as sources of COVID-19 information. HCPs had adequate knowledge, positive attitude and good prevention practice towards COVID-19. Being a Nurse, having < 5 years of working experiences, using social media and television and or radio were factors associated with good prevention practice. Thus, developing HCP’s professional carrier through training opportunities, sharing experiences and using verified information sources are crucial to better improve COVID-19 prevention practice.

## Introduction

Coronavirus disease 2019 (COVID-19) is caused by a novel human coronavirus called severe acute respiratory syndrome coronavirus 2 (SARS-CoV-2) [1,2]. The first case of COVID-19 was identified in Wuhan, Hubei province, Chin by the end of the year 2019 [2,3]. Within a month of its appearance, the disease has spread dramatically around the world. Because of this nature, it was declared as a public health emergency that deserves international attention. Following its declaration as a global pandemic by the World Health Organization (WHO) again, all countries are called for collaborative efforts to combat the threat caused by this virus [4,5]. Worldwide, the severe disturbance of COVID-19 has been well noticed in terms of the daily confirmed cases and attack rates, associated mortalities and disrupted social and community lives [6,7]. Even though the largest spread of COVID-19 has been occurred in Europe and America, African countries were amongst the latest to be affected by the disease [7–10]. The huge comorbidities, low socio-economic condition, poor quality of care and the limited access to healthcare facilities would make the scene to be significant in the African situation [7,8].

Healthcare providers (HCPs) are at an increased risk of getting COVID-19 as a result of their front-line works [11]. Based on the experience from the 2003 SARS outbreak, one–fifth of the global burden of SARS cases occurred among healthcare workers (HCWs) [12]. Similarly, many HCPs have been infected with COVID-19 and others lost their lives in the current pandemic. As of 27 July 2020, the WHO estimated that more than 10% of the total COVID-19 cases worldwide occurred among HCPs with marked variations in countries across the world [10,13]. When compared with others, the sources of infection to COVID-19 in HCPs are plural i.e. from the community and working places. The HCPs are also predominant transmitters of infection to families, patients and the community [11–13]. The transmission of COVID-19 among HCPs is associated with overcrowding, lack of isolation room facilities, and unhygienic environmental conditions. However, this is likely coupled with the fact that some HCWs have inadequate knowledge of infection prevention practices [14].

Indeed, protection of HCPs and their working environment are relevant aspects in pandemic responses. This requires that HCPs must have up-to-date knowledge and optimistic attitude towards the many aspects of the pandemic. As well, preventive health behaviors of the HCPs have a direct COVID-19 effect on their and community health. These health behaviors are acquired, maintained, or changed with the help of the motivation and skills of HCPs [15,16]. In addition, increasing the awareness and preventive behavior of HCPs with continuous updates about COVID-19 is relevant [17,18]. Health behaviors of HCPs can influence prevention and control actions implemented in response to the pandemic [15]. Since HCPs are vital in the fight against COVID-19 pandemic, their prevention behavior takes a lion share of containing the infection among themselves and reducing the spread to others. This notably depends on their knowledge, attitude and practice in dealing with this highly transmissible virus [19]. As part of the pandemic response therefore, exploring HCP’s knowledge, attitude and prevention practice is very important. These helps to notice deficiencies in COVID-19 understanding, related perceptions and prevention practices and thereby justify the significance to train frontline vulnerable HCPs on IPC skills [19,20].

Studies in various settings have indicated that there are huge differences in terms of the knowledge, attitude, and practice (KAP) of HCWs in the fight against the pandemic. Different factors like socio-demographic, knowledge and attitude were also identified to be associated with COVID-19 prevention practice [8,21–24]. Despite the fact that HCPs play a central role in the response to COVID-19, to our knowledge there is very limited information on the KAP of HCPs towards COVID-19. Also, few studies assessed HCPs prevention health behaviors during COVID-19 [25–27]. Particularly, data on the Ethiopian situation regarding the subject matter are lacking. However, such data are important in designing evidence-based pandemic prevention and control interventions for this vulnerable group. Identifying the factors influencing prevention practice in a given health system is also a critical step to reduce infection prevention barriers. Therefore, the purpose of this study was to assess the knowledge, attitude, and practice and associated factors of prevention practice towards COVID-19 among healthcare providers in Amhara region, northern Ethiopia.

## Materials and methods

### Study design, setting and population

A multi-center cross-sectional study was conducted from September to October 2020 among HCPs working in selected public health facilities in Southwest Amhara region, Ethiopia. Amhara region is the second-largest region in the country and its capital city is Bahir Dar city. The region has a total of twelve administration Zones. Located in the Southwest of Amhara region, East Gojjam Zone has 22 districts and three Town administrations. Our study was undertaken in four public hospitals and thirteen health centers in Southwest of Amhara region. All HCPs aged 18 years and above in the selected public health facilities were included in the study. The name of selected public health facilities and their location is given in the table below **(**Table 1).

**Table 1:** Name and location of selected public health facilities in Amhara region, Ethiopia, 2020.

Ser. No	Name and Type of HF	Town/city	District
1	Debre Markos Comprehensive Specialized Hospital (DMCSH)	Debre Markos	Debre Markos
2	Bichena Primary Hospital (BPH)	Bichena	Enemay
3	Bibugn Primary hospital (BPH)	Guha-Tsion	Bibugn
4	Merto-Lemariam Primary Hospital (MLPH)	Merto-Lemariam	Enebse-Sarmidir
5	Amanuel health center	Amanuel	Machakel
6	Degasign health center	Amanuel	Machakel
7	Kidamin health center	Amanuel	Machakel
8	Debrekelem health center	Amanuel	Machakel
9	Merto-Lemariam health center	Merto-Lemariam	Enebse-Sarmidir
10	Maraki health center	Merto-Lemariam	Enebse-Sarmidir
11	Guha-Tsion health center	Guha-Tsion	Bibugn
12	Bibugn health center	Guha-Tsion	Bibugn
13	Debre Markos health center	DM town	Debre Markos
14	Hidase health center	DM town	Debre Markos
15	Wuseta health center	DM town	Debre Markos
16	Bichena health center	Bichena city	Enemay
17	Enemay health center	Bichena city	Enemay

### Sample size and sampling procedure

A sample size for this study was calculated using a single population proportion formula with the assumption of 50% of HCPs had COVID-19 prevention practice since no previous study on COVID-19 related KAP, with a 5% margin of error at a 95% confidence level, i.e. N = (1.96)^2^ *0.5*0.5/(0.05)^2^ = 384 and after adding a 10% non-response rate, the final sample size was 422. From all districts found in Southwest of Amhara region, five districts namely Debre Markos, Machakel, Enemay, Enebse-sarmidir and Bibugn were selected using simple random sampling method. The estimated number of HCPs working in each district was obtained from the Regional Health Bureau. Based on the obtained information, the sample size for each district was proportionally allocated. The public health facilities found in the selected districts were first stratified as hospital and health centers. Then, the hospitals found in the four selected districts were obtained purposefully while the health centers in all selected districts were selected using lottery method. Finally, the study subjects were proportionally allocated for each selected health facilities. The HCPs in the selected health facilities were selected through simple random sampling procedure using the registered list of all HCPs provided from the human resource office of each selected public health facilities.

### Data collection tool and procedure

Data related to HCP’s KAP and socio-demographic characteristics were collected using a structured and pre-tested self-administered questionnaire. The questionnaire was adapted and modified from previously published studies [28,29] and guidelines suggested by WHO related to COVID-19 IPC measures [19]. The questionnaire was initially prepared in English (S1 English version of the questionnaire) then translated to the local Amharic language (S2 Amharic version of the questionnaire) and translated back to English language to ensure its consistency and accuracy. Even though HCPs might read a questionnaire which is prepared in English language, it was hard to conclude that all of them would understand and interpret it in the same way. With the aim of conveying similar message and obtaining an anonymous response from all participants, the questionnaire used in this study was translated in to local Amharic language. The questionnaire was pre-tested on 5% of the study participants before the actual data collection period in health facilities which were not selected for the main study. Based on findings from the pretest, the clarity, appropriateness and redundancy of questions were revised and corrected accordingly. The questionnaire was divided into four sections. The first section assessed the socio-demographic characteristics of HCPs. The second section assessed the respondents’ COVID-19 related knowledge. The third section assessed the respondents’ COVID-19 related attitude and the fourth section assessed the respondents’ COVID-19 prevention practice.

### Study variables and measurement

The main outcome variable in the study was COVID-19 prevention practice while socio-demographic and COVID-19 related characteristics were the independent variables for this study.

### Socio-demographic measures

Socio-demographic information was collected and included age, sex, profession, facility type, years of working experience and sources of COVID-19 related information. *Age* of HCPs was divided in two categories: 1) <30 years and 2) ≥30 years. The cut-off for age was based on previous studies used to define HCPs as younger and older age groups [30,31]. *Working experience*, the number of years served as a health professional, was divided in two categories: 1) <5 years and 2) ≥5 years. The cut-off for working experience was based on prior studies conducted in Pakistan and Saudi Arabia [32,33]. Membership in COVID-19 taskforce in this study described whether HCPs were active members of a committee established to run COVID-19 prevention and related activities or not, and was categorized as ‘Yes’ and ‘No’.

### Knowledge, attitude and practice

KAP related to COVID-19 were assessed using a total of 26 items/questions (13 knowledge, 7 attitude and 6 practice) which were adapted from the work of Taghrir et al. and Roy et al. [34,35]. The different number of items and value scoring systems used to categorize KAP were then modified from the work of Olum et al., Bloom et al. and Goni et al. [8,36,37]. Accordingly, a cut-off ≥ 80% (≥ 10 points out of 13), ≥90% (≥ 12 points out of 14), and ≥75% (≥ 5 points out of 6) was used to determine adequate knowledge, positive attitude, and good prevention practice, respectively.

Knowledge about COVID-19 was based on a 13-item scale that assessed HCP’s understanding about the causative agent, mode of transmission, high risk groups, clinical manifestation as well as prevention and treatment of COVID-19. Each knowledge question had a possible response of *“True”*, *“False”* and “*Don’t know”*. Then, the correct answer (*True*) was coded as 1, while the wrong answer (*False /don’t know*) was scored as 0 during analysis. Accordingly, the total score ranged from 0–13, with an overall greater score indicated adequate knowledge. Based on the modified Bloom’s cut-off point, a healthcare provider who scored ≥80% of the correct knowledge questions (≥10 points out of 13) was considered as having *“*adequate knowledge*”* and who scored <80% (<10 points out of 13) was considered as having “inadequate knowledge.” The reason for using an 80% cut off value for knowledge was by considering that majority of HCPs would attend training courses with respect to COVID-19 and they were also more likely to receive COVID-19 related information from a variety of information sources [36,37].

Attitude toward COVID-19 was based on a 7-item scale that assessed HCP’s attitude towards treatment, infection control procedure and related information about COVID-19. The response of each statement was indicated on a 3-point Likert scale as follows: 2(“*Agree”)*, 1(“*Undecided*”) and 0(“*Disagree*”). Accordingly, the total score ranged from 0–14, with an overall greater score indicated positive attitude. Based on the modified Bloom’s cut-off point, a healthcare provider who scored ≥90% of the favorable attitude statements (≥12 points out of 14) was considered as having *“*positive attitude*”* and who scored <90% (<12 points out of 14) was considered as having “negative attitude.” The reason for using a 90% cut off value for attitude was by considering the uncontrolled nature of the pandemic and HCP’s concerns of becoming infected, particularly given the shortage of personal protective equipment (PPE) [8,36,37].

COVID-19 related practice was based on a 6-item scale that assessed HCP’s use of PPE and other counter measures. Each practice-related question was responded as “*Yes regularly*”, *“Not at all”* and “*occasionally*”. Accordingly, the total score ranged from 0–6, with an overall greater score indicated good prevention practice. Based on the modified Bloom’s cut-off point, a healthcare provider who scored ≥75% of the correct practice questions (≥5 points out of 6) was considered as having *“*good prevention practice*”* and who scored <75% (<5 points out of 6) was considered as having “poor prevention practice.” The reason for using a 75% cut off value for practice was by considering the seriousness of the COVID-19, and the study participants are healthcare providers to whom the prevention practice is mandatory to keep themselves and their families safe from COVID-19 and be a role model to their patients and the rest of the community [8,36].

### Data processing and analysis

Data were checked, coded, and entered into Epi Info version 7.0 and exported to SPSS version 25.0 for analysis. Descriptive statistics such as frequency, percentages, mean, and standard deviation were computed to summarize categorical and numerical data. Bloom’s modified cut-off point was used to determine adequate knowledge (≥80%), positive attitude (≥90%) and good prevention practice (≥75%) (36). Bivariable logistic regression analysis was initially done to identify the candidate independent variables which had association with COVID-19 prevention practice. Then, all independent variables having a p-value of < 0.2 in the bivariable analysis were transferred to the multivariable analysis to identify significantly associated factors of prevention practice. Model fitness for the final model was checked using Hosmer and Lemeshow goodness of fit. Statistical significance was determined at a p-value of < 0.05 and the presence of association was described by using odds ratio (OR) with their 95% confidence interval (CI).

### Ethics statement

Ethical clearance and approval was obtained from the Ethical Review committee of College of Health Science, Debre Markos University. A formal letter of cooperation was obtained from each district administration and health facility. After the aim of the study was clearly explained, both written and verbal consent were obtained from all study participants. The consent form documented the aims, nature, and procedure of the study. The privacy and confidentiality of information was also strictly guaranteed by all data collectors and investigators.

## Results

### Socio-demographic and COVID-19 related characteristics of study participants

Of the total 422 HCPs approached, 410 HCPs responded to the self-administered interview, giving a response rate of 97%. The mean age of the participants was 28.9 years (SD± 5.88) and the age range was from 20–52 years old. Nearly two-thirds of the participants were male and aged below 30 years. Most of the study participants were Nurses (42.9%), worked in hospitals (61%) and served for < 5 years (58.2%). Television and/or radio (42%) and social media (35.1%) were identified by participants as the major sources of information about COVID-19. In addition, 386 (94.1%) participants heard about COVID-19. Over half (54.6%) of the HCPs attended formal training on COVID-19 and 182 (44.4%) of them were members of the COVID-19 taskforce (Table 2).

**Table 2:** Socio-demographic characteristics of participants among health care providers in Amhara region, Ethiopia, 2020.

Characteristic		Number (%)
Age group (years)	<30	264 (64.4)
	≥30	146 (35.6)
Sex	Female	140 (34.1)
	Male	270 (65.9)
Profession	Physician	50 (12.2)
	Nurse	176 (42.9)
	Health officer	52 (12.7)
	Pharmacist	64 (15.6)
	Others*	68 (16.6)
Health care providers’ health facilities	Health center	160 (39.0)
	Hospital	250 (61.0)
Working experience (years)	<5	239 (58.3)
	≥5	171 (41.7)
Source of information about COVID-19	Social media e.g., Facebook	144 (35.1)
	Television and/ or radio	172 (42.0)
	Others**	94 (22.9)

Others*-Midwife, Laboratory staff, Anesthetist and Radiologist.

Others**-Workshop and seminar, Poster and leaflet, Colleagues and official government website.

### Knowledge of HCPs about COVID-19

The finding showed that nearly nine in ten HCPs (89.8%) had adequate knowledge about COVID-19. More than 90% of the participants were aware of the causative agent, high risk population groups, severity and fatality of COVID-19, and the care needed for COVID-19 symptomless individuals. Most of the participants (87.3%) identified fever, dry cough, and shortness of breath as the major symptoms of the COVID-19 infection. The majority of HCPs (85.9%) mentioned washing hands with soap and water and wearing a face mask as vital ways to prevent COVID-19. Over three-fourths of the participants (79.5%) mentioned as COVID-19 can be transmitted by droplets and close contact. Slightly below two-thirds of the HCPs reported the correct incubation period (2–14 days), and only 14.9% HCPs reported the current presence of a vaccine for COVID-19 (Table 3).

**Table 3:** COVID-19 related knowledge of participants among health care providers in Amhara region, Ethiopia, 2020.

Knowledge about COVID-19		Number (%)
The causative agent of COVID-19 is a virus	False	10 (2.4)
	True	400 (97.6)
COVID-19 can be severe and leads to death if not tread early	False	30 (7.3)
	True	380 (92.7)
The incubation period of COVID-19 virus is 2–14 days	False	154(37.6)
	True	256 (62.4)
COVID-19 can be transmitted by droplets and close contact	False	84 (20.5)
	True	326 (79.5)
Antibiotics are the first-line treatment for COVID-19	False	378 (92.2)
	True	32 (7.8)
Fever, cough and shortness of breath are major symptoms of COVID-19	False	52 (12.7)
	True	358 (87.3)
Currently there is a vaccine for COVID-19	False	349 (85.1)
	True	61 (14.9)
Polymerase chain reaction (PCR) is vital for the diagnosis of COVID-19	False	140 (34.1)
	True	270 (65.9)
Chronically ill and elderly people are at highest risk of COVID-19	False	24 (5.9)
	True	386 (94.1)
COVID-19 patients can develop severe acute respiratory illness	False	2 (.5)
	True	408 (99.5)
Influenza vaccine also gives protection from COVID-19	False	356 (86.8)
	True	54 (13.2)
COVID-19 symptomless individuals also needs care	False	40 (9.8)
	True	370 (90.2)
Hand washing and wearing face masks are vital to prevent COVID-19	False	58 (14.1)
	True	352 (85.9)

### Attitudes of HCPs towards COVID-19

As indicated by the findings from this study, the majority of respondents (94.4%) had a positive attitude toward COVID-19. The vast majority of respondents strongly agreed on mandatory quarantine (93.2%), the use of gowns and gloves while giving care (87.8%), dissemination of COVID-19 related scientific information (82.4%), and active involvement of HCPs in infection control programs (81.5%). Most (74.1%) HCPs strongly believed that particular and sufficient care must be given for a patient with COVID-19. Only 29% of HCPs strongly believed that COVID-19 transmission can be prevented through practicing widely recommended official measures (Table 4).

**Table 4:** COVID-19 related attitude of participants among health care providers in Amhara region, Ethiopia, 2020.

Attitude toward COVID-19		Number (%)
COVID-19 confirmed patients need to be kept in mandatory quarantine	Strongly agree	382(93.2)
	Agree	28(6.8)
It is good to use gowns, gloves and mask while giving care for patients	Strongly agree	360 (87.8)
	Agree	50(12.2)
HCPs have to disseminate COVID-19 related scientific information	Strongly agree	338(82.4)
	Agree	72(17.6)
HCPs shall take the initiative in COVID-19 infection control program	Strongly agree	334(81.5)
	Agree	76(18.5)
A patients with COVID-19 needs particular and sufficient care	Strongly agree	304(74.1)
	Agree	106(25.9)
It is possible to prevent COVID-19 via practicing official measures	Strongly agree	119(29.0)
	Agree	291(71.0)

### COVID-19 prevention practice of HCPs

Based on the findings of this study, nearly four-fifths (79.5%) of the HCPs had good prevention practices towards COVID-19. About 82.9% of the participants regularly covered their mouth and nose while sneezing and 82.4% of them wore face masks in crowded situations. Similarly, three-fourths (75.9%) of the HCPs reported to use a hand sanitizer regularly and about 71% of them regularly gave health education about COVID-19 for their patients/ clients (Table 5).

**Table 5:** COVID-19 prevention practices of participants among health care providers in Amhara region, Ethiopia, 2020.

COVID-19 prevention practices		Number (%)
Do you use hand sanitizer before and after handling patients?	Occasionally	99 (24.1)
	Yes regularly	311 (75.9)
Do you use tissue to cover while sneezing or coughing?	Occasionally	70 (17.1)
	Yes regularly	340 (82.9)
Do you wash hands frequently using water and soap?	Occasionally	91 (22.2)
	Yes regularly	319 (77.8)
Do you avoid touching your mouth/nose/eye?	Occasionally	72 (17.6)
	Yes regularly	338 (82.4)
Do you use face mask in crowded situations?	Occasionally	72 (17.6)
	Yes regularly	338 (82.4)
Do you educate patients/clients about COVID-19?	Occasionally	119 (29.0)
	Yes regularly	291 (71.0)

### Factors associated with COVID-19 prevention practice

From the multivariable logistic regression analysis being a Nurse in profession, having < 5 years of working experience, using social media and radio and/or television as sources of COVID-19 information had a significant association with HCP’s COVID-19 prevention practice. HCPs who were Nurses in their profession were two times (AOR = 2.13, 95% CI = 1.13–3.99) more likely to have good COVID-19 prevention practice than Physicians. HCPs who had < 5 years of working experience were 46% times more likely to have good COVID-19 prevention practice than those who had ≥ 5 years of working experience (AOR = 0.46; 95% CI = 0.24, 0.86). The odds of having good COVID-19 prevention practice was six and four times higher among HCPs who used social media and radio and/or television (AOR = 6.20; 95% CI = 2.33, 16.51; AOR = 4.03; 95% CI = 1.56, 10.38), respectively when compared with those who used other sources of COVID-19 information (Table 6).

**Table 6:** Factors associated with COVID-19 prevention practice of participants among health care providers in Amhara region, Ethiopia, 2020.

Variables	Categories	Practice		Crude Odds Ratio (COR) (95%CI)	Adjusted Odds Ratio (AOR) (95%CI)
		Good	Poor		
Sex	Male	208 (50.7)	62 (15.1)	0.32 (0.16–0.61)	0.72 (0.32–1.63)
	Female	128 (31.2)	12 (3.0)	1	1
Age	>30	125 (30.5)	21 (5.1)	0.67(0.39–1.16)	0. 80 (0.44–1.46)
	<30	211 (51.5)	53 (12.9)	1	1
Occupation	Nurses	152 (37.1)	24 (5.9)	3.56(1.34–5.19)	2.13 (1.13–3.99)*
	Health officers	2 (0.5)	50 (12.2)	0.02(0.01–46.99)	0. 17 (0. 07–1.59)
	Pharmacist	54 (13.2)	10 (2.4)	3.04(0.96–5.28)	1.78(0.65–4.89)
	Others	48 (11.7)	20 (4.8)	1.35 (0.34–1.61)	0.55(0.19–1.61)
	Physician	32 (7.8)	18 (4.4)	1	1
Experience	< 5 years	185 (45.1)	54 (13.2)	2.20(1.26–3.84)	0.46(0.24–0.86)*
	≥ 5 years	151 (36.8)	20 (4.9)	1	1
Source of information	Social media	128 (31.2)	16 (3.9)	4.97(2.55–9.66)	6.20(2.33–16.51)*
	Radio & TV	150 (36.6)	22 (5.4)	4.23(2.29–7.79)	4.03(1.56–10.38)*
	Others	58 (14.1)	36 (8.8)	1	1
Knowledge	Adequate	305 (74.4)	63 (15.4)	1.72.(0.82–3.59)	0.76 (0.28–2.06)
	Poor	31 (7.6)	11 (2.6)	1	1

## Discussion

This study assessed KAP among HCPs and identified factors associated with COVID-19 prevention practice in Southwest Amhara region, Ethiopia. Accordingly, the proportion of adequate knowledge, positive attitude and good prevention practice towards COVID-19 among HCPs was 89.8%, 94.4%, and 79.5%, respectively. Furthermore, this study found that being a Nurse in profession, having < 5 years of working experience, using social media and radio and/or television as sources of COVID-19 information had a significant association with HCP’s COVID-19 prevention practice.

In the current study, nearly nine in ten (89.8%) HCPs had adequate knowledge about COVID-19. This finding was almost comparable with prior studies conducted in Greece, Pakistan, and China where 88.28%, 89% and 93.2% of HCPs had sufficient knowledge of COVID-19 respectively [21,32,38]. Our finding was higher than the studies conducted elsewhere in Ethiopia. For example, 70% of HCPs in Addis Ababa and 74% in northern Ethiopia had good knowledge of COVID-19 as compared to the current finding [26,27]. This difference might be due to variation in the nature of the study. The former studies were about knowledge of HCPs on the overall infection prevention practice while our study is knowledge about COVID-19 which is a current and timely concern. Thus, there is a repeated exposure to COVID-19 related information since the coronavirus pandemic is being widely communicated in the world. As well, it has been talked via social and mass media to inform HCPs and the population at large. Moreover, our result is also much higher than the studies conducted in Nigeria and Iran where 56.5% and 56% of HCPs were knowledgeable about COVID-19 respectively [23,34]. This variation could be due to the difference in the study area, geographic coverage, and the nature of questions used to assess the knowledge variable.

Our study revealed that majority of HCPs (94.4%) had a positive attitude toward COVID-19. This finding was in line with the studies conducted in Pakistan and Vietnam where the majority of HCWs had a positive attitude towards COVID-19 [32,39]. Although the majority of participants reported high level of positive response for each items of attitude, only 29% of the HCPs in our study strongly agreed that COVID-19 transmission can be prevented through practicing widely recommended precaution measures. This result suggested that only a small number of respondents were very much confident of the recommended COVID-19 prevention measures. This might be due to the fact that almost half of HCPs were not attending COVID-19 related training and the training may be inadequate for those who trained on COVID 19. Based on the result and information presented here, it was informative that training courses and trusts worthy attitudinal inspirations need to be placed given the seriousness of the disease.

Our study showed that about four-fifth of HCPs had good COVID-19 prevention practice. This result was comparable with a study conducted in Uganda where 74% of HCPs had a good practice towards COVID-19 [8]. This finding was higher than a study conducted in Ethiopia which reflected that 62% of HCWs had good preventive practice [27]. The possible explanation could be the difference in sample size, method of data collection and coverage of the study area. In contrast, this finding was lower than findings from Pakistan and China in which 88.7% and 89.7% of HWs had a good practice regarding COVID-19 respectively [32,38]. The presence of big differences in socio-economic and healthcare system might explain the variations of findings in these countries. Because these situations could greatly affect the availability of PPE, IP policies, access to and adequacy of trainings, information sources and awareness levels of HWs [30,31]. Majority of the HCPs in our study were regularly exercising the recommended infection prevention and control practices. These included covering eye and mouth with a tissue while sneezing/coughing, avoidance of touching eyes and mouth and wearing a face mask when in crowded situation and when in contact with patients. These were very essential practices to prevent the transmission of COVID-19 from HCPs to patients and among HCPs themselves. However, only 71% of HCPs admitted giving health education for clients about COVID-19 on regular basis. This might be attributed to the high work load of HCPs that would make them to be busy to provide valuable information on COVID-19.

In this study, the results for prevention practice were slightly lower than those for knowledge (79.5% vs 89.8%). Meaning, despite a higher proportion of adequate knowledge among HCPs in this study, the proportion of good prevention practice was slightly lower. This finding was in contrast with earlier studies which indicated that HWs with better knowledge would practice more preventive measures [36]. However, HWs better knowledge on IP strategies depend on their information sources, access to training and presence of IP guidelines among others [8,36,37]. In general, having sufficient knowledge may reflect the wider dissemination of information about COVID-19 through different media. Nevertheless, about 77% of HCPs in our study seemed to use social and news media as the main sources of information about COVID-19. This finding was however a significant concern given the credibility and reliability of the information obtained from these sources. Although information from these sources has had a positive impact on the knowledge of HCPs, HCPs might not practice all what they know considering that misinformation and disinformation may mislead to wrong practice. This finding was in contrast with studies conducted in Iran and Vietnam where the website of WHO and ministry of health were used as the main sources of information about COVID-19 [39,40]. This suggested that HCPs are more likely to have good prevention practice if they are encouraged to use official websites as an essential and credible source of information.

Some of the significant factors associated with COVID-19 prevention practice were profession of HCPs, working experience and sources of COVID-19 related information. The current study affirmed that HCPs who were Nurses in their profession were five times more likely to have good COVID-19 prevention practice as compared to Physicians. This finding was supported by a prior study conducted globally, but in contrast with a study conducted in Uganda [8,31]. This might be due to the chance of better refresher trainings, personal commitment and follow-up. Working experience was one of the factors that were significantly associated with COVID-19 prevention practice among HCPs in this study. HCPs with less than five years of working experience were more likely to have good prevention practice as compared to their counterparts. Those with less than five years of working experience might receive repeated training about COVID-19 and its prevention and they might be as effective in watching the right ways of pandemic prevention mechanisms demonstrated via trusted sources or websites. HCPs that had less than five years of work experience were 46% times more likely to have good prevention practice than HCPs who had five and above years of working experience. This is linked to the ongoing training provided for HCPs with limited years of experience. This was in contrast with earlier studies [21,26].

Our study showed that source of information has a significant association with HCP’s COVID-19 prevention practice. The likelihood of good COVID-19 prevention practice was about four times more likely among HCPs who used Radio and TV as source of COVID-19 information as compared to those who used other sources. Similarly, other previous studies reported good COVID-19 prevention practice in HCPs who used news media like Radio and TV [36]. Similarly, participants who used social media as a source of information had about six times more chance of having good COVID-19 prevention practice as compared to those who used other sources. This finding was in line with studies conducted globally and in Vietnam [31,39].

### Limitations

The study had some limitations. Firstly, no standardized tool for assessing KAPs on COVID-19 has been validated previously. We have however adapted and modified a previously published tool used to assess KAP among HCPs [25,31]. The questions have been organized from WHO and CDC guidelines and reports on COVID-19 [34]. Especially, the questions used to measure the knowledge of HCPs about COVID19 (including the available treatment options and supportive cares) are not sufficient enough. Secondly, only HCPs in governmental health facilities were participated in the survey and the results of this study may not reflect the KAPs of HCPs in the whole region. Thirdly, data were collected using self-administered techniques so that the validity of data may be affected in some extent. Since it is a one-time study, it shared the limitations of a cross-sectional study to show cause-effect relationships between the independent and the outcome variables.

## Conclusions

This study concluded that HCPs had adequate knowledge, positive attitude and good prevention practice regarding the COVID-19 pandemic. Avoidance of touching eyes and mouth and wearing face masks regularly were the preventive measures that HCPs were most likely to comply with. Profession, working experience and sources of COVID-19 information had showed a positive significant association with COVID-19 prevention practices. Likewise, being a Nurse, having < 5 years of working experience, using social media and television and or radio as information sources were the factors significantly associated with good COVID-19 prevention practice. Thus, developing HCPs’ professional carrier through training opportunities, sharing experiences and using verified information sources are crucial to better improve COVID-19 prevention practice. Also, building the skills of HCPs and updating their knowledge are required to reduce the risk of acquiring COVID-19 infection.

## Supporting information

S1 Text English version questionnaire. (DOCX)

S2 Text Amharic version questionnaire. (DOCX)

S1 Data SPSS dataset. (SAV)
