# Supplementary material for: Knowledge, attitude, practice, and factors associated with prevention practice towards COVID-19 among healthcare providers in Amhara region, northern Ethiopia: A multicenter cross-sectional study
Source: PLOS Glob Public Health. 2022 Apr 11;2(4):e0000171. doi: 10.1371/journal.pgph.0000171 (PMC10021359; doi:10.1371/journal.pgph.0000171)
Supplement: S1 Text — (DOCX) [file pgph.0000171.s001.docx]

**Title: COVID-19 prevention practice and associated factors among healthcare providers in Amhara region, northern Ethiopia: A multicenter cross-sectional study.**

**Principal Investigator: Wassachew Ashebir Kebede**

Department of Public Health, College of Medicine and health sciences, Debre Markos University, Ethiopia

**Information sheet**

Coronavirus disease 2019 (COVID-19) is a global health emergency caused by severe acute respiratory syndrome coronavirus 2 (SARS-CoV-2). It has become a public health threat in Ethiopia causing around 5 thousand new cases and 80 deaths as of 23 June 2020. In this case, healthcare providers (HCPs) are vital in the fight against COVID 19 pandemic and are particularly vulnerable to the disease as a result of their front-line works. More than 10% of the total COVID 19 cases worldwide occur among HCPs with marked variations in countries across the world. When compared with others, the sources of infection to COVID-19 for HCPs are plural i.e. from the community and working places. The HCPs are also predominant transmitter of infection to families, patients and the community. Preventive health behaviors of the community, family and individuals have a direct COVID-19 effect on their health.

Inadequate knowledge and poor practice of HCPs can determine their potential of prevention towards COVID-19. This will make them to have limited preventive behavior and further spread of the disease to others. Thus, being updated with valid information is crucial to HCPs to manage their patients safely and keep themselves and their families’ free safe from acquiring COVID-19 infection. They also need have better access to proper protective equipment at work places. Therefore, the purpose of this study was to assess COVID-19 preventive practice and associated factors among HCPs in northern Ethiopia. This will be critical to make decisions on improving infection prevention practices in health facilities through accessing the required materials and trainings.

Data for this study will be collected using structured and pre-tested self-administered questionnaire. As you know, it is highly secured and no any personal identifier is needed after data collection. No one other than the investigator will have access to your data. You are randomly selected by chance from all lists of HCPs in the region. You will get nothing incentives because of your participation except being part of the COVID-19 prevention and evidence generation. The information you provide will assist the researchers in rapidly develop response to protect the healthcare providers and enabling healthcare providers to prevent and mitigate COVID-19 outbreak. Participation is fully voluntary including withdrawing from the participation. It will take only 10 - 15 minutes to complete. If you are volunteer to be part of this study, please read, sign and send the following consent form. If you need further clarity, please contact the investigator; Wassachew Ashebir Kebede at Email: [ashebirwase@gmail.com](mailto:ashebirwase@gmail.com)

By clicking the next button, I read and gave my consent to take part in this study.

*Required

**Statement of Consent**

I have been asked to participate in the research study. The study has been explained well to me. I understand what the study means to me including what I (the participant) have to go through while in the study. I have had an opportunity to ask questions about the study and have been answered in the best way for me to understand. If there are any other questions that I have to ask later, I will freely approach the study representatives whose contact I have been provided with. I also understand that my participation is voluntary, my data will be confidential with no use of any personal identifies. I have told as my consent can be withdrawn any time in the meantime without any precondition. Thus, I have accepted the offer to be part of this study using my signature.

Participant ID. _________________ Signature_______________ date______________

**Questionnaires**

**Instructions**

1. For each section, respondents are well come to choose all the possible choices/ more than one answer.
2. A required field on each page must be completed to pass to the next question or page. [*****Required]
3. Adequate glove? Mask, sanitizer, etc means: If you have no shortage to your daily use of protective equipment and you have not missed to use since the occurrence of COVID-19 and no a shortage message from your facility.
4. The questionnaire comprised of four sections: A) Socio-demographic characteristics of HCPs that consisted of 9 questions. B) COVID-19 related knowledge consisted of 13 questions that ask about its causative agent, fatality, incubation period, mode of transmission, signs and symptoms, availability of vaccines and treatment regimens, at high risk groups and preventive measures. C) COVID-19 related attitude (7 questions). 4). COVID-19 preventive practice of health care providers from getting COVID-19 infection (6 questions).

**NB**: To each section, you can select more than one or all the possible choices to the questions

| **A** | **Socio-demographic related questions** | |
| --- | --- | --- |
| **Sr.N.** | **Questions** | **Response** |
| 1 | Age in years | _______________ |
| 2 | Your sex | □ Male □ Female |
| 3 | Your profession | □ Physician □ Nurse □ Health officer □ Pharmacist □ Other____________ |
| 4 | The type of health facility you work with | □ Health center □ Hospital |
| 5 | What is your source of information regarding COVID-19 | □ Social Media  □ Television and or radio  □ Seminars & workshops  □ Posters & Pamphlets  □ Seniors & Other Colleagues  □ Official Government website |
| 6 | Are you a member of COVID-19 taskforce | □Yes □ No |
| 7 | How long have you been working there in years | _____________ |
| 8 | Did you attend training related to COVID 19 in this year | □Yes □ No |
| 9 | Have you heard that COVID 19 is occurring worldwide | □Yes □ No |
| **B** | **COVID-19 related knowledge questions** | |
| 1 | The causative agent of COVID −19 is a virus | □No □Yes |
| 2 | Coronavirus infection could be fatal | □No □Yes |
| 3 | The incubation period of COVID- 19 virus is 2-14 days | □No □Yes |
| 4 | Respiratory droplets and close contact with infected person are major transmission modes of COVID-19 | □No □Yes |
| 5 | Antibiotics are the first-line treatment for COVID-19 | □No □Yes |
| 6 | Fever, dry cough and shortness of breath are the major symptoms of COVID-19 | □No □Yes |
| 7 | Currently there is a vaccine for COVID-19 | □No □Yes |
| 8 | Polymerase chain reaction (PCR) is a diagnostic tool to COVID-19 virus infection | □No □Yes |
| 9 | Patients with underlying chronic diseases like diabetes and hypertension are at a higher risk of COVID-19 infection | □No □Yes |
| 10 | COVID-19 patients can develop severe acute respiratory illness | □No □Yes |
| 11 | Influenza vaccine also gives protection from COVID-19 | □No □Yes |
| 12 | Special caution must be taken if someone develop symptoms and signs suggestive of COVID-19 | □No □Yes |
| 13 | Washing hands with soap and water, and using face masks can help to prevent COVID-19 | □No □Yes |
| **C** | **COVID-19 related attitude questions** | |
| 1 | Gowns, gloves, mask and goggles must be used when dealing with COVID-19 patients | □ Strongly agree □ Agree □ Neutral  □Disagree □ Strongly disagree |
| 2 | COVID-19 patients should be kept in isolation | □ Strongly agree □ Agree □ Neutral  □ Disagree □ Strongly disagree |
| 3 | Intensive and emergency treatment should be given to COVID-19 diagnosed patients | □ Strongly agree □ Agree □ Neutral  □ Disagree □ Strongly disagree |
| 4 | Prevalence of COVID-19 can be reduced by active participation of HCPs in the hospital infection control program | □ Strongly agree □ Agree □ Neutral  □ Disagree □ Strongly disagree |
| 5 | Any related information about COVID-19 should be disseminated among HCPs | □ Strongly agree □ Agree □ Neutral  □ Disagree □ Strongly disagree |
| 6 | Transmission of COVID-19 infection can be prevented by using universal precautions given by WHO, CDC | □ Strongly agree □ Agree □ Neutral  □ Disagree □ Strongly disagree |
| 7 | HCPs must acknowledge themselves with all the information about COVID-19 | □ Strongly agree □ Agree □ Neutral  □ Disagree □ Strongly disagree |
| **D** | **COVID-19 preventive practice** **questions** | |
| 1 | Do you use hand sanitizer? | □ Not at all □ Yes regularly |
| 2 | Do you cover your nose and mouth with a tissue during sneezing or coughing?? | □ Not at all □ Yes regularly |
| 3 | Do you use frequent handwashing with water and soap /or alcohol-based bund rub sterilizer as per recommended? | □ Not at all □ Yes regularly |
| 4 | Do you avoid touching your eyes, nose or mouth as far as you can? | □ Not at all □ Yes regularly |
| 5 | Do you routinely wear a facemask or shields at work and outside working places | □ Not at all □ Yes regularly |
| 6 | Do you educate your patient about COVID-19? | □ Not at all □ Yes regularly |
