# Supplementary material for: Knowledge, attitude, practice, and factors associated with prevention practice towards COVID-19 among healthcare providers in Amhara region, northern Ethiopia: A multicenter cross-sectional study
Source: PLOS Glob Public Health. 2022 Apr 11;2(4):e0000171. doi: 10.1371/journal.pgph.0000171 (PMC10021359; doi:10.1371/journal.pgph.0000171)
Supplement: S2 Text — (DOCX) [file pgph.0000171.s002.docx]

**ደብረማርቆስ ዩኒቨርሲቲ**

**ጤና ሳይንስ ኮሌጅ**

**የህብረተሰብ ጤና ት/ት ክፍል**

**የምርምር/ ጥናት ማብራሪያ እና የስምምነት መስጫ ቅፅ**

ጤና ይስጥልኝ። ስሜ_________________________ ይባላል።

እዚህ የመጣሁት በደብረማርቆስ ዩኒቨርሲቲ የጤና ሳይንስ ኮሌጅ የሕብረተሰብ ጤና
ት/ት ክፍል መምህራን በቡዲን ለሚያጠኑት ጥናት መረጃ ሰብሳቢ ሆኜ ነዉ። የጥናቱ ዋና ዓላማ በተመረጡ የምስራቅ ጎጃም ጤና ተቋማት የሚሰሩ ጤና ባለሙያዎች ሰለ ወቅታዊ የኮሮና ቫይረስ በሽታ ያላቸዉን እዉቀት፣አመለካከት አና ተግባር ማጥናት ነው። እርሶ በዚህ ጥናት አንዲሳተፉ ተመርጠዋል። በዚህ ጥናት ላይ በመሳተፍዎ መጠይቁን ለመሙላት የሚጠይቀዉን የተወሰኑ ደቂቃዎች ከማጥፋትዎ በስተቀር የሚደርስብዎት ምንም ጉዳት የለም፤ ነገር ግን አንዳንድ ጥያቄዎች ግላዊ ቢመስሉም ለጥናቱ አስፈላጊ ናቸዉ፡፡ በተጨማሪ በዚህ ጥናት ስለተሳተፉ የሚያገኙት ክፍያ የለም ምንም እንዃን ከጥናቱ ዉጤት እንደ ዜጋ ሊያገኙት የሚችሉት ጥቅም ሊኖር ቢችልም፡፡ በዚህ ጥናት መጠይቅ ተሳታፊ የሚሆኑት በፍቃደኝነት ነው። ያለመሳተፍ ወይም በመሀል የማቆም መብት አለዎት ፤ ቢሆንም ግን የእርስዎ ትክክለኛ መረጃ ለዚህ ጥናት ጠቃሚ ነዉ፤ የሚሰጡን መረጃ ሚስጥራዊነቱ የተጠበቀ እና ለጥናታዊ ተግባር ብቻ የሚዉል እና ለማንም የማይገለጽ ይሆናል፡፡
በጥናቱ ለመሳተፍ ተስማምተዋል?

1) አዎ ተስማምቻለሁ 2) አልተስማማሁም

**በዚህ ጥናት ለመሳተፍ ፈቃደኛ ከሆኑ፣ በቀጣይ ገፅ ለተሰጡት አምስት ክፍል መጠይቆች ተገቢዉን መልስ ከፊት ለፊት በተቀመጠላቸዉ አማራጭ መሰረት ይሙሉ**

**አመሰግናለሁ!**

**የአማርኛ መጠይቅ**

| **የጤና ባለሙያዎች ኮሮና ቫይረስን በተመለከተ ስላላቸዉ እዉቀት፣አመለካካት፣ተግባር እንድሁም የበሽታ መከላከል ትግበራ ለማወቅ የተዘጋጀ**  **Code__________________** | | | |  |
| --- | --- | --- | --- | --- |
| **ክፍል አንድ: የጤና ባለሙያዎች አጠቃላይ መረጃ** | | | | |
| **S.no** | **Variables** | **Categories** | **Skip** | |
| **101** | **እድሜ** | **----------- ዓመት** |  | |
| **102** | **ፆታ** | 1. **ወንድ 2. ሴት** |  | |
| **103** | **የስራ ልምድ** | **----------- ዓመት ---------- ወር** |  | |
| **104** | **ሙያ** | 1. **ሃኪም 2. ጤና መኮንን 3. ነርስ 4. ፋርማሲስት 5. ሌላ ካለ-------------** |  | |
| **105** | **አሁን የሚሰሩበት የጤና ተቋም አይነት** | **1. ጤና ጣቢያ**  **2. ሆስፒታል** |  | |
| **106** | **ኮሮና ቫይረስን በተመለከተ፣ መረጃ የሚያገኙበት ዘዴ** | **1. ማህበራዊ ሚድያ**  **2. ሬድዮ እና ቴሌቪዠን**  **3. ሰሚናር እና ወርክሾፕ**  **4. ፖስተር እና በራሪ ወረቀት**  **5. ከስራ ባልደረባና ጓደኞች**  **6. ከታወቀ የመንግስት ድህረ ገፅ**  **7. ሌላ ካለ--------------------------** |  | |
| **107** | **የኮሮና ቫይረስ መከላከል ግብረ ሀይል አባልነት** | **1. አዎ 2. አይደለሁም** |  | |
| **108** | **ኮሮና ቫይረስ በመላዉ አለም አየተሰራጨ ያለ አድስ ቫይረስ ነዉ** | **1. አዉቃለሁ**  **2. አላዉቅም** |  | |
| **109** | **ኮሮና ቫይረስን በሚመለከት ስልጠና ወይም ዉይይት ላይ መሳተፍ** | **1. ተሳትፊያለሁ**  **2. አልተሳተፍኩም** |  | |
| **ክፍል ሁለት: የጤና ባለሙያዎች ኮሮና ቫይረስን በተመለከተ ስላላቸዉ እዉቀት የቀረበ መጠይቅ** | | | | |
| **S.n** | **Variables** | **Category/response** | **Skip** | |
| **201** | **ኮሮና (COVID-19) በቫይረስ አማካኝነት የሚመጣ በሽታ ነዉ** | **1. አዎ 2. አይደለም** |  | |
| **202** | **የኮሮና ቫይረስ በሽታ የህክምና ክትትል ካልተደረገ እስከ ሞት ያደርሳል** | **1. አዎ 2. አይደለም** |  | |
| **203** | **አንድ ሰዉ ለኮሮና ቫይረስ ከተጋለጠ ቡሃላ ምልክቶችን እሰከሚያሳይ ድረስ ከ2 እስከ 14 ቀናት ሊወስድ ይችላል** | **1. አዎ 2. አይደለም** |  | |
| **204** | **የኮሮና ቫይረስ የመጀመሪያ ደረጃ ህክምና ፍቱን መድሃኒት መስጠት ነዉ** | **1. አዎ 2. አይደለም** |  | |
| **205** | **ትኩሳት፣ ደረቅ ሳል እና ለመተንፈስ መቸገር ወይም የትንፋሽ ማጠር የኮሮና ቫይረስ ምልክቶች ናቸዉ** | **1. አዎ 2. አይደለም** |  | |
| **206** | **የኮሮና ቫይረስ የመከላከያ ክትባት በአሁኑ ሰዓት በስራ ላይ ዉሏል** | **1. አዎ 2. አይደለም** |  | |
| **207** | **Polymerase chain reaction (PCR) የኮሮና ቫይረስን ለመመርመር ይረዳል** | **1. አዎ 2. አይደለም** |  | |
| **208** | **ተጓዳኝ የጤና እክል ለምሳሌ የስኳር በሽታ፣ ከፍተኛ ደም ግፊት ወዘተ ያለባቸዉ ሰዎች ለከባድ የኮሮና ህመም የተጋለጡ ናቸዉ** | **1. አዎ 2. አይደለም** |  | |
| **209** | **የኮሮና ቫይረስ ሰዎች በሚቀራረቡ እና በሚጠጋጉ ጊዜ በሳል ፡ በማስነጠስ እና በመነካካት ይተላለፋል** | **1. አዎ 2. አይደለም** |  | |
| **210** | **እጅን በአግባቡ በውሃና ሳሙና ደጋግሞ መታጠብ የኮሮና ቫይረስ ስርጭትን ይከላከላል** | **1. አዎ 2. አይደለም** |  | |
| **211** | **የኮሮና ቫይረስ ተጠቂዎች ከባድ የመተንፈሻ አካላት ችግር ሊገጥማቸዉ ይችላል** | **1. አዎ 2. አይደለም** |  | |
| **212** | **እንድ ሰዉ ትኩሳት፣ ማሳል፣የትንፋሽ ማጠር ፣እና ለመተንፈስ፣ መቸገር ምልክቶችን ካሳየ ልዩ ጥንቃቄ ሊደረግ ይገባል** | **1. አዎ 2. አይደለም** |  | |
| **213** | **የኢንፉሌንዛ ቫይረስ ክትባት የኮሮናቫይረስ በሽታንም ለመከላከል ይረዳል** | **1. አዎ 2. አይደለም** |  | |
| **ክፍል ሶስት: የጤና ባለሙያዎች ኮሮና ቫይረስን በተመለከተ ስላላቸዉ አመለካከት የቀረበ መጠይቅ** | | | | |
| **S.n** | **Variables** | **Category or response** | **Skip** | |
| **301** | **ለኮሮና ቫይረስ ተጠቂዎች ድጋፍና ህክምና ሲደረግ እንደ ግዋን, ገላቭ, ማስክ እና የመሳሰሉት መጠበቂያዎች ሊደረጉ ይገባል** | **1. በጣም እስማማለሁ 2. እስማማለሁ**  **3. አይመለከተኝም 4. አልስማማም**  **5. በጣም አልስማማም** |  | |
| **302** | **የኮሮና ቫይረስ በምርመራ የተገኘባቸዉ ሰዎች በለይቶ ማቆያ ዉስጥ መደረግ አለባቸዉ** | **1. በጣም እስማማለሁ 2. እስማማለሁ**  **3. አይመለከተኝም 4. አልስማማም**  **5. በጣም አልስማማም** |  | |
| **303** | **በፅኑ ህመም ላይ ያሉ የኮሮና ቫይረስ ተጠቂዎች አፋጣኝና፣ ያላሰለሰ ህክምናና ክትትል ሊደረግላቸዉ ይገባል** | **1. በጣም እስማማለሁ 2. እስማማለሁ**  **3. አይመለከተኝም 4. አልስማማም**  **5. በጣም አልስማማም** |  | |
| **304** | **የጤና ባለሙያዎችን በሆሰፒታል የበሽታ መከላከል ፕሮገራሞች በማሳተፍ የኮሮና ቫይረስ ስርጭትን መቀነስ ይቻላል** | **1. በጣም እስማማለሁ 2. እስማማለሁ**  **3. አይመለከተኝም 4. አልስማማም**  **5. በጣም አልስማማም** |  | |
| **305** | **ማንኛዉም የኮሮና ቫይረስን የሚመለከት መረጃ በሁሉም የጤና ባለሙያዎች በኩል ተደራሽና ለሌሎች የሚሰራጭ መሆን ይኖርበታል** | **1. በጣም እስማማለሁ 2. እስማማለሁ**  **3. አይመለከተኝም 4. አልስማማም**  **5. በጣም አልስማማም** |  | |
| **306** | **የአለም ጤና ድርጅትና ሲዲሲ በሚያወጡት ሁሉንአቀፍ የጥንቃቄ መርህ የኮሮና ቫይረስ ስርጭትን መከላከል ይቻላል** | **1. በጣም እስማማለሁ 2. እስማማለሁ**  **3. አይመለከተኝም 4. አልስማማም**  **5. በጣም አልስማማም** |  | |
| **307** | **የጤና ባለሙያዎች ስለኮሮና ቫይረስ ለሚኖራቸዉ መረጃና ጥንቃቄ ሊመሰገኑ ይገባል** | **1. በጣም እስማማለሁ 2. እስማማለሁ**  **3. አይመለከተኝም 4. አልስማማም**  **5. በጣም አልስማማም** |  | |
| **ክፍል አራት: የጤና ባለሙያዎች ኮሮና ቫይረስን በተመለከተ ስላላቸዉ ተግባር የቀረበ መጠይቅ** | | | | |
| **S.n** | **Variables** | **Category/response** | **Skip** | |
| **401** | **እጅዎን በየጊዜዉና ቶሎቶሎ ሲታጠቡ፣ሳሙናና የእጅ ማፅጃ ሳኒታየዘሮችን ይጠቀማሉ?** | **1. አዎ 2. የለም** |  | |
| **402** | **በሚያስሉበት እና በሚያስነጥሱበት ጊዜ ክርንዎን በማጠፍ፣ አፍ እና አፍንጫዎን በሶፍት /ሌላ ነገር ይሸፍናሉ?** | **1. አዎ 2. የለም** |  | |
| **403** | **ሲያስሉና ሲያስነጥሱ የተጠቀሙበትን ሶፍት ወዲያዉኑ በአግባቡ አስወግደው እጅዎን ይታጠባሉ?** | **1. አዎ 2. የለም** |  | |
| **404** | **በተቻለዎት መጠን፣አይንዎን፤ ጆሮዎን እና አፍዎን ላለመንካካት ይሞክራሉ?** | **1. አዎ 2. የለም** |  | |
| **405** | **ሰው የተሰበሰበት ቦታ፤ ትላልቅ ስብሰባዎችን እና ማህበራዊ ክንውኖች ላይ የፊት ጭንብል ይጠቀማሉ?** | **1. አዎ 2. የለም** |  | |
| **406** | **ኮሮና ቫይረስን በተመለከተ፣ አገልግሎት ለሚሰጧቸዉ ሰዎች ትምህርት ይሰጣሉ?** | **1. አዎ 2. የለም** |  | |

**አመሰግናለሁ**
